# Bridging the gap in pediatric cancer rehabilitation care: a multi-perspective survey study

**DOI:** 10.3389/fped.2026.1870844

**Published:** 2026-07-07

**Authors:** Filip Jevič, Anna Vašinová, Aleš Rak, Andrea Kašparová, Marie Ohnisková, Adelaida Dizonová, Kristýna Pospíšilová

**Affiliations:** 1 Department of Rehabilitation and Sports Medicine, Second Faculty of Medicine, Charles University, Motol and Homolka University Hospital, Prague, Czechia; 2 Second Faculty of Medicine, Charles University, Prague, Czechia; 3 Department of Gender and Sociology, Institute of Sociology of the Czech Academy of Sciences, Prague, Czechia

**Keywords:** access gap, Czech Republic, multi-perspective survey, pediatric oncology, physiotherapy, rehabilitation

## Abstract

**Background:**

Rehabilitation is a fundamental pillar of supportive care in pediatric oncology, yet its delivery faces significant systemic barriers. Data on rehabilitation accessibility and provider readiness in Central Europe remain limited.

**Objectives:**

This study examined the rehabilitation needs of pediatric oncology patients aged 0–19 years both during and after treatment — including accessibility, barriers, and stakeholder readiness — from the perspectives of parents, physiotherapists, and rehabilitation physicians.

**Methods:**

Three parallel questionnaire surveys were conducted among parents of children with cancer (*N* = 394), physiotherapists (*N* = 318), and rehabilitation physicians (*N* = 85), and analyzed using descriptive statistics, binary logistic regression, and inductive thematic analysis of open-ended responses.

**Results:**

While 63.7% of parents reported their child required rehabilitation during active treatment, only 53.3% ultimately received it — an unmet need rate of 10.4% that rose to 13.3% after treatment completion. Lymphoma and spinal cord tumor patients showed the highest unmet need rates across both phases. Rehabilitation proved a long-term necessity: 29.5% of children required therapy for more than six months during treatment, rising to 47.6% post-treatment. A critical education gap was identified: 80% of physiotherapists and 74% of rehabilitation physicians rated their graduation-level knowledge as insufficient, a deficit persisting in nearly half of professionals in current practice. Qualitative analysis revealed structural barriers including informational deficits, insufficient regional infrastructure, and professional uncertainty regarding patient safety. Despite these hurdles, 66% of physiotherapists and 54% of physicians expressed interest in joining a specialized competence network (RehaSÍŤ).

**Conclusion:**

The findings confirm a substantial gap between chronic rehabilitation demand and healthcare system capacity. Professional reluctance reflects insufficient training rather than lack of motivation. Establishing targeted educational programs and a structured competence network such as RehaSÍŤ may be key to ensuring equitable access to high-quality supportive care.

## 1 Introduction

While the survival rate for childhood cancer in high-income countries now exceeds 80% (1), pediatric oncology is facing a hidden crisis: up to 90% of survivors develop at least one chronic health condition, and many suffer from lifelong functional and motor deficits that significantly impair their quality of life (2, 3). Despite the fact that late effects of anticancer treatment—such as chemotherapy-induced peripheral neuropathy, cancer-related fatigue, and neuromotor impairments—profoundly impact functional mobility and physical performance (4), comprehensive rehabilitation in pediatric oncology remains structurally neglected in real-world clinical practice (5).

The International Pediatric Oncology Exercise Guidelines clearly state that physical activity and structured exercise are safe for children and adolescents with cancer and provide substantial health benefits (6). Numerous clinical trials and reviews have robustly demonstrated the efficacy of targeted rehabilitation in improving cardiorespiratory fitness, muscle strength, and physical performance (7). Guidelines emphasize that rehabilitation should begin as early as possible after the diagnosis is established or after surgery (8). The rehabilitation plan must be carefully tailored to the patient's individual diagnosis, planned cytostatic treatment protocol, and the broader psychosocial and family context (8). In practice, however, care is often fragmented, inaccessible, and unsystematic, with significant disparities frequently arising between specialized oncology centers and peripheral community facilities (9, 10). While current literature predominantly focuses on the clinical efficacy of these interventions, there is a critical knowledge gap regarding their actual accessibility and systemic implementation in real-world practice.

Recently, attention has shifted from merely demonstrating the efficacy of exercise to addressing these structural barriers in community-based care (11). Systematic reviews across allied health professions consistently identify a triad of implementation barriers — insufficient professional knowledge, lack of time, and limited access to resources — as the primary factors preventing rehabilitation professionals from translating evidence-based guidelines into routine clinical practice (12, 13). Recent initiatives in Western Europe and Australia have further highlighted the need to bridge the gap between specialized hospital centers and community-based primary care. For instance, preliminary needs assessments for the developing *KinderOncoNet* in the Netherlands revealed that while parents strongly prefer rehabilitation close to home, community pediatric physiotherapists often lack disease-specific knowledge (14). Similarly, the ReActivate program in Australia aims to coordinate care for adolescent survivors (15). However, evidence regarding these systemic barriers remains largely confined to Western models. The Czech Republic represents a significantly underexplored context for such research. As a Central European country characterized by different healthcare financing and organizational structures — where rehabilitation physicians traditionally serve as primary coordinators of care — these implementation barriers may be further compounded by limited interdisciplinary integration and the absence of standardized community-based care pathways. While the pan-European long-term follow-up network PanCare provides a vital platform for coordinated survivorship interventions (16, 17), national data on systemic barriers are urgently needed to drive policy changes.

Therefore, the aim of the present cross-sectional study was to quantify the “access gap” and identify the systemic and educational barriers to rehabilitation in the Czech Republic. By utilizing a unique triangulation of data from three key stakeholder groups—parents of pediatric oncology patients, physiotherapists, and physicians—this study assesses the availability of rehabilitation during active cancer treatment and throughout survivorship. The findings aim to serve as an evidence-based foundation for the “RehaSÍŤ” project, a national multidisciplinary network designed to integrate standardized rehabilitation care pathways into pediatric oncology, providing a model potentially generalizable to other regions with similar healthcare structures.

## 2 Methods

### 2.1 Study design

Within the RehaSÍŤ project, three questionnaires were developed for key stakeholder groups involved in the rehabilitation care of pediatric oncology patients: parents of children with cancer, physiotherapists, and rehabilitation physicians. This represents the first study of its kind in the Czech context to examine this issue using a quantitative design and data triangulation across three stakeholder groups. Data from the three questionnaire surveys were collected simultaneously from May 12 to June 30, 2025, using the anonymous online survey platform LimeSurvey. Participation was voluntary and informed consent was obtained electronically prior to survey completion. The study was approved by the Ethics Committee of the University Hospital Motol and 2nd Faculty of Medicine, Charles University in Prague (ref. no. EK 39/25).

### 2.2 Questionnaires

The questionnaires for parents and physiotherapists were developed on the basis of a survey instrument used in a Dutch needs assessment of parents of children with cancer and community pediatric physiotherapists (14). The original Dutch questionnaires were provided by the authors and subsequently translated into Czech by a professional translator; accuracy was verified by an independent back-translation. Both questionnaires included closed-ended and open-ended questions and additional items were included where necessary. Given the specific coordinating role of rehabilitation physicians within the Czech healthcare system, a separate questionnaire for this group was developed by a rehabilitation physician with clinical experience in pediatric oncology, largely modeled on the physiotherapist version.

All three questionnaires underwent pilot testing, and representatives of the target groups participated in a focus group aimed at discussing their content validity, clarity, and comprehensibility. A total of 34 participants were included in pilot testing: 7 parents, 1 childhood cancer survivor, 9 rehabilitation physicians, 11 physiotherapists, and 3 representatives of non-profit organizations, in addition to 3 members of the research team. Each participant completed the questionnaire corresponding to their professional or parental role; the survivor and non-profit representatives completed the parent version. Following completion, content, clarity, and comprehensibility were discussed among all participants and the research team.

The questionnaires were subsequently reviewed by an expert in quantitative sociological research methodology from the Czech Academy of Sciences, and final adjustments were made based on this consultation. The estimated completion time was approximately 10 min for parents and 20 min for healthcare professionals.

### 2.3 Participants

Eligible participants were physiotherapists, parents of children with cancer, and rehabilitation physicians from across the Czech Republic. For parents, inclusion criteria included being the parent of a child who was currently undergoing or had previously undergone oncological treatment, regardless of the time elapsed since treatment completion. For healthcare professionals, inclusion criteria included active professional practice as a physiotherapist or rehabilitation physician with experience working with pediatric patients in any clinical setting, regardless of prior experience with pediatric oncological patients.

Participants were recruited through parent organizations and foundations, professional associations, and healthcare institutions. Invitations were distributed via email, social media platforms, and printed posters displayed in selected oncology and rehabilitation departments. A convenience sampling approach with voluntary self-selection was used.

### 2.4 Statistical methods

Data from three questionnaire surveys presented above (parents, rehabilitation physicians and physiotherapists) were analyzed using IBM SPSS Statistics version 25.0. Questionnaires with substantial missing data were excluded from the analysis. Descriptive statistics were used to summarize the main findings for all three target groups. For categorical variables, data are presented as counts and percentages, and differences between groups were assessed using the *χ*² (chi-square) test at 5% significance level.

Binary logistic regression (BLR) was subsequently applied to examine factors associated with awareness, lack of access to rehabilitation care (parental questionnaire), and physiotherapists’ willingness to accept pediatric oncology patients during active treatment (physiotherapists' questionnaire). This approach enabled us to analyze associations between variables such as diagnosis, age of the child, regional differences or physiotherapist characteristics, while controlling for potential confounding effects.

To ensure adequate cell sizes and statistical stability, some variables were recategorized. Diagnosis was categorized into five groups: lymphoma (reference category), acute leukemia, Central nervous system (CNS) tumors, osteosarcoma, and other. CNS tumors comprised brain tumors and spinal cord tumors, which were combined into a single category due to their clinical similarity and limited sample size in the spinal cord subgroup. Child's age was grouped into three categories: 0–6 years (reference), 7–12 years, and 13–19 years, collapsing the original five age categories used in descriptive analyses. Primary treatment centers were coded as Brno (reference), Motol, and others.

### 2.5 Qualitative analysis of open-ended questions

Qualitative data from the survey were analysed using exploratory, inductive thematic analysis, following the six-phase framework (18). The analysis focused on participants' written responses to open-ended questions selected for their clinical relevance. Specifically, these included barriers parents faced when rehabilitation care was not provided during or after oncological treatment, including limitations related to regional accessibility of care, as well as barriers to providing pediatric oncological rehabilitation from the perspective of physiotherapists and rehabilitation physicians, and the perceived benefits and added value of the RehaSÍŤ network for all stakeholder groups. The full list of open-ended questions is provided in Supplementary Material (Supplementary Table S1).

After familiarization with the data, ATLAS.ti software was used to support the coding process and organisation of the data. The coding was conducted towards the descriptive and semantic end of the analytic spectrum, capturing the explicit content of participants' responses (19). Codes were refined through multiple readings of the dataset and organised into groups, forming the basis of themes and subthemes. Coding was conducted by a single researcher; to enhance qualitative trustworthiness, a second researcher reviewed a subset of responses and codes as a peer debriefing to confirm the validity of the initial coding. The structure and interpretation of the themes were reviewed and discussed within the research team to ensure accurate representation of the data.

All qualitative responses were collected in Czech. Selected verbatim quotations are presented in the Results section to illustrate the identified themes, introduced where applicable by the label “Selected quotations:”. Quotations were selected to represent the breadth of responses within each theme rather than their frequency alone.

## 3 Results

**Figure 1:**
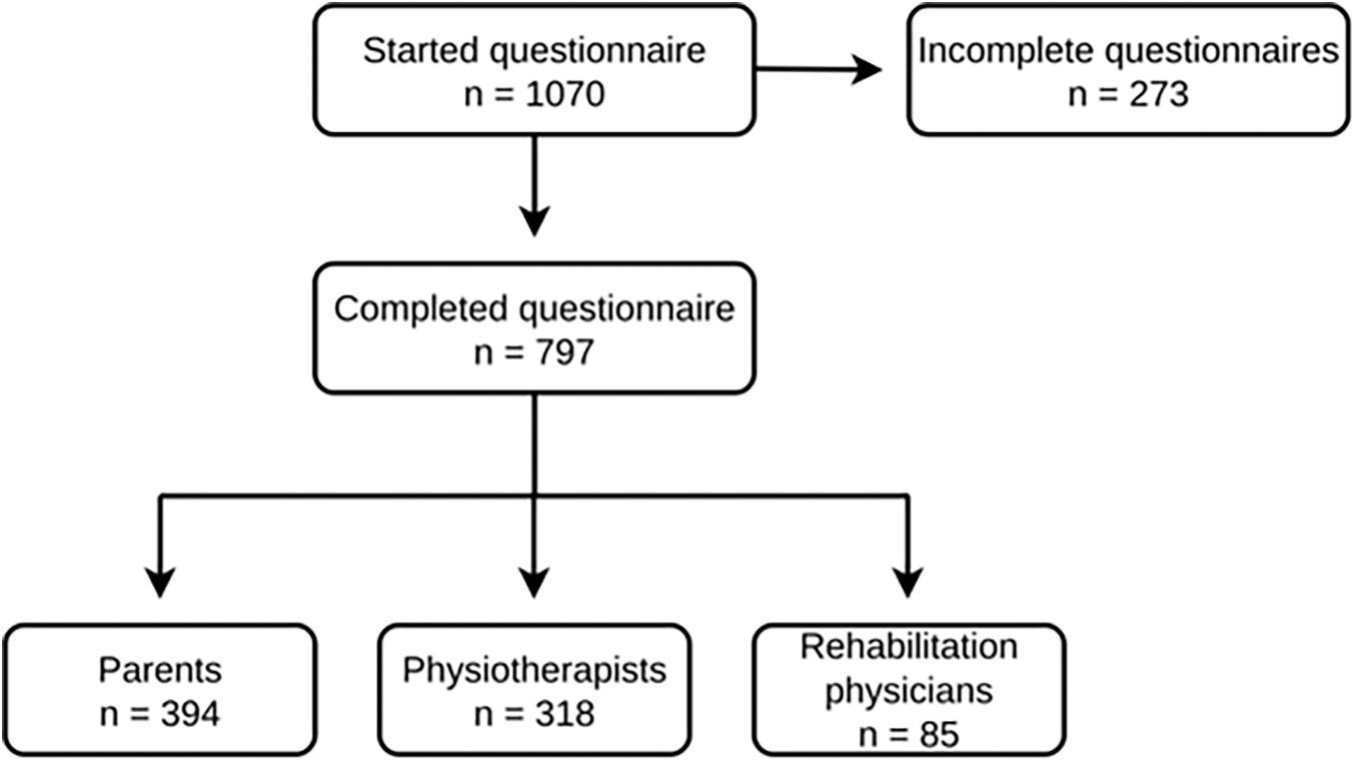
Participant flow diagram. Of the 1,070 individuals who initiated one of three parallel questionnaires — developed separately for parents of children with cancer, physiotherapists, and rehabilitation physicians — 273 were excluded due to incomplete responses, yielding a final analytic sample of 797 participants (394 parents, 318 physiotherapists, and 85 rehabilitation physicians).

A total of 1,070 individuals initiated the survey; after excluding 273 incomplete responses, 797 participants were included in the final analysis (394 parents, 318 physiotherapists, and 85 rehabilitation physicians) (Figure 1).

### 3.1 Sociodemographic and professional characteristics

Given the nature of the data collection, full representativeness of the sample cannot be guaranteed; however, good coverage across all regions of the Czech Republic was achieved. For analytical purposes, regions were categorized into Prague (the capital city), the rest of Bohemia, the South Moravian Region (with a major center for cancer treatment in Brno), and the rest of Moravia, as regional differences were assumed. For 65.5% of children, the place of primary treatment was Motol University Hospital, for 25.4% it was University Hospital Brno, and for 9.1% it was other hospitals.

The distribution of oncological diagnoses is consistent with the national epidemiological profile of childhood cancer in the Czech Republic (20, 21), with Acute lymphoblastic leukemia (ALL) and CNS tumors comprising the two largest diagnostic groups (Table 1). All pediatric age groups and treatment phases are represented across the dataset, supporting the generalizability of findings across the continuum of pediatric oncology care.

**Table 1:** Sociodemographic and clinical characteristics of the parental sample.

Parents	Number	Percentage (%)
Region		
Prague	71	18.0
Rest of Czechia	216	54.8
South Moravian region	40	10.2
Rest of Moravia	67	17.0
Place of primary treatment		
Motol University Hospital	258	65.5
University Hospital Brno	100	25.4
Other	36	9.1
Highest education of parent		
Primary school	14	3.6
Secondary without diploma	39	9.9
Completed secondary education	148	37.6
University degree	189	48.0
Undisclosed	4	1.0
Diagnosis of the child		
ALL	104	26.4
Lymphoma	42	10.7
CNS tumor	70	17.8
Spinal cord tumor	12	3.0
Bone tumor	30	7.6
Others	136	34.5
Age of child at diagnosis		
Less than 1 year	32	8.1
1–3 years	98	24.9
4–6 years	96	24.4
7–12 years	87	22.1
13–19 years	81	20.6
Phase of treatment		
Intensive treatment	67	17.0
Maintenance treatment	71	18.0
Treatment finished ˂1year	62	15.7
Treatment finished >1 and ˂ 4 years	148	37.6
Treatment finished >4 years	46	11.7

Data are presented as absolute frequencies (n) and valid percentages (%). The geographical distribution of respondents covers all administrative regions of the Czech Republic. ALL, acute lymphoblastic leukemia; CNS, central nervous system;.

The gender distribution of participating healthcare professionals is consistent with national workforce data from the Czech Statistical Office, which recorded 87% female physiotherapists in 2022 (22). Comparable population-level data on rehabilitation physicians are not systematically published in the Czech Republic, precluding direct benchmarking for this group. Nonetheless, the sample demonstrates broad diversity across several dimensions relevant to generalizability: professionals were distributed across all regions of the Czech Republic, represented a wide range of workplace settings and specializations, and encompassed a broad spectrum of clinical experience, from early-career professionals to those with more than 20 years of practice. Together, these characteristics suggest that the sample captures a diverse cross-section of the Czech rehabilitation workforce, though the use of convenience sampling precludes formal claims of population representativeness. Full professional characteristics of both groups are presented in Table 2.

**Table 2:** Professional characteristics and work settings of participating healthcare providers.

	Physiotherapists		RHB physicians	
	Number	Percentage (%)	Number	Percentage (%)
Gender				
Male	30	9.4	15	17.6
Female	287	90.3	69	81.2
Undisclosed	1	0.3	1	1.2
Region of primary workplace				
Prague	71	22.3	17	20.0
Rest of Czechia	126	39.6	40	47.1
South Moravian region	30	9.4	11	12.9
Rest of Moravian	91	28.6	17	20.0
Primary working place setting (MR)				
Outpatient clinic (insurance-covered)	183	40.3	33	38.8
Private practice (out-of-pocket)	113	24.9	6	7.1
General hospital	35	7.7	29	34.1
University hospital	46	10.1	20	23.5
Inpatient rehabilitation center/Medical spa	19	4.2	13	15.3
Day rehabilitation center	17	3.7	5	5.9
Home-based practice (mobile physiotherapy)	17	3.7	0	0.0
Other	24	5.3	5	5.9
Duration of praxis				
<1 year	46	14.5	0	0.0
1–3 years	34	10.7	7	8.2
4–6 years	44	13.8	8	9.4
7–9 years	43	13.5	14	16.5
10–20 years	80	25.5	29	34.1
>20 years	71	22.3	27	31.8
Specialisation (MR)				
Physical and Rehabilitation Medicine	–	–	77	90.6
Pediatric Neurology	–	–	1	1.2
Pediatrics	–	–	12	14.1
Orthopedics	–	–	3	3.5
Other	–	–	11	12.9

Data are presented in numbers and percentages (%) unless otherwise indicated. Percentages may not sum to 100 due to rounding or because some items allowed multiple responses. Items allowing multiple responses are indicated in the table (MR). For the item “Primary working place setting”, several administrative regions were combined into broader regional categories for presentation purposes. Categories of specialization of rehabilitation physicians and primary workplace setting were based on the Czech healthcare system context. RHB physicians, rehabilitation physicians;

### 3.2 Parental perspectives

#### 3.2.1 Awareness of rehabilitation entitlement

A fundamental prerequisite for accessing rehabilitation is parental awareness of its availability. However, only 51.0% of parents reported being explicitly informed about their child's eligibility for rehabilitation services, while 27.7% stated they had never received this information and 21.3% could not recall receiving it. This informational deficit stands in sharp contrast to parental expectations: 64.2% of parents scored 8–10 on a 10-point scale when asked whether they expected to be referred to a pediatric physiotherapist with specific oncology expertise at their primary treatment center, with 50.8% indicating absolute agreement (score 10).

Associations between parental awareness of rehabilitation entitlement and clinical/demographic variables (diagnosis, child's age at diagnosis, and primary treatment center) were examined using binary logistic regression, with awareness of rehabilitation eligibility coded as the binary outcome (awareness = 1, otherwise = 0).

Binary logistic regression revealed that the likelihood of parental awareness varied significantly according to the child's diagnosis and primary treatment center (Table 3). Compared to parents of children with lymphoma (reference category), parents of children with CNS tumors were eight times more likely to report having been informed, followed by parents of children with osteosarcoma, who reported five times higher awareness and parents of children with acute leukemia, who reported 4.5 times higher awarenesscompared to the reference category. Child's age at diagnosis was not a significant predictor. Treatment at Motol University Hospital was associated with twice the likelihood of awareness compared to treatment in Brno, suggesting significant institutional variability in the provision of rehabilitation-related information.

**Table 3:** Binary logistic regression model for predicting parental awareness of rehabilitation eligibility.

Predictor	Category	*β*	S.E.	*p*-value	OR	95% CI for OR
Diagnosis	Lymphoma (ref.)			0.000		
	ALL	1.509	0.511	0.003	4.521	(1.66–12.30)
	CNS tumor	2.081	0.532	0.000	8.016	(2.82–22.79)
	Bone tumors	1.691	0.606	0.005	5.423	(1.65–17.82)
	Other	0.628	0.472	0.184	1.874	(0.74–4.74)
Age at diagnosis	0–6 years (ref.)			0.951		
	7–12 years	0.067	0.343	0.845	1.069	(0.55–2.09)
	13–19 years	−0.062	0.378	0.869	0.940	(0.45–1.97)
Treatment center	Brno (ref.)			0.020		
	Motol	0.810	0.295	0.006	2.248	(1.26–4.01)
	Other	0.275	0.507	0.587	1.317	(0.49–3.56)
Constant		−1.082	0.516	0.036	0.339	(0.12–0.93)

*N* = 374 parents; ref., reference category; β, regression coefficient; S.E., standard error; OR, odds ratio; CI, confidence interval; statistical significance set at *p* < 0.05. Omnibus test of model coefficients is statistically significant; Nagelkerke R^2^ = 15%.

#### 3.2.2 Unmet rehabilitation needs

The distribution of children who required and received rehabilitation, did not require rehabilitation, and required but did not receive rehabilitation differed significantly between the treatment and post-treatment phases (chi-square test was statistically significant).

**Figure 2:**
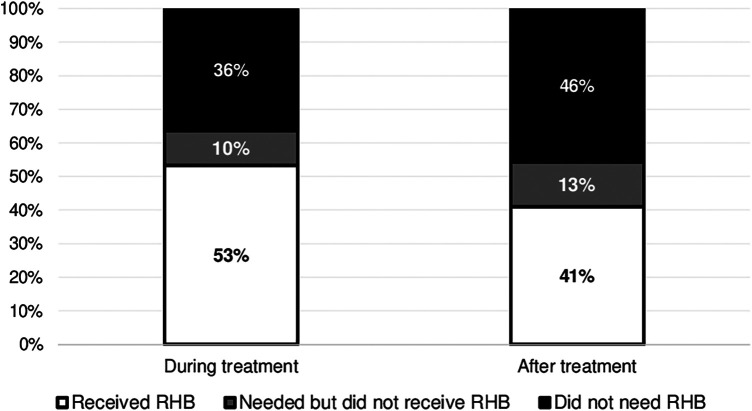
Overview of rehabilitation access during and after oncological treatment. Stacked bar chart showing parental-reported rehabilitation access during active oncological treatment (*n* = 394) and after treatment completion (*n* = 256). Percentages are calculated from the total parent cohort for each phase. A statistically significant difference was found between rehabilitation care by treatment phase (*χ*²(2) = 9.378, *p* = 0.009, Cramér's *V* = 0.12). RHB, rehabilitation.

Of the 394 parents, 63.7% reported that their child required rehabilitation during active oncological treatment, yet only 53.3% ultimately received it. Overall, 10.4% of parents (*n* = 41) reported that their child required but did not receive rehabilitation during the treatment phase, indicating that approximately one in ten children with cancer did not receive needed rehabilitation. Following treatment completion, this proportion increased to 13.3% (*n* = 34 of 256 respondents), suggesting that the gap between need and provision widens after treatment completion. The overall pattern of rehabilitation access across both phases is illustrated in Figure 2.

**Figure 3:**
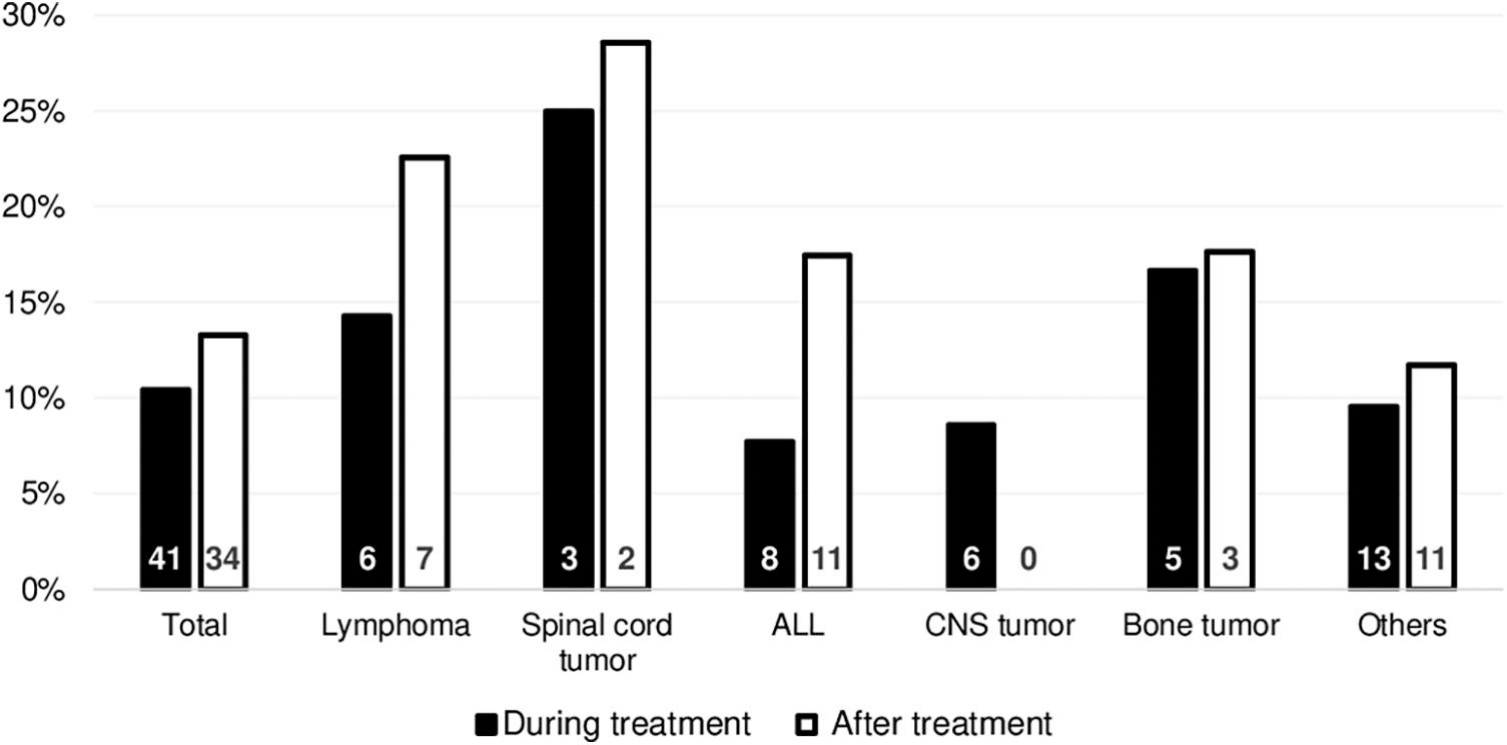
Unmet rehabilitation needs among pediatric oncology patients by primary diagnosis. Bars represent the proportion of patients within each diagnostic group who required but did not receive rehabilitation, calculated from all patients per diagnostic group (during treatment: *n* = 394; after treatment: *n* = 256). Numbers within bars indicate absolute counts. ALL, acute lymphoblastic leukemia; CNS, central nervous system.

When stratified by primary diagnosis, spinal cord tumor and lymphoma patients showed the highest rates of unmet need across both phases, while ALL patients showed the lowest rate during treatment and CNS tumor patients reported no unmet needs post-treatment (Figure 3). Given the small absolute numbers in several subgroups, these percentages should be interpreted with caution.

Binary logistic regression was used to examine predictors of unmet rehabilitation needs. In this analysis, only parents of children who required rehabilitation care were included (*N* = 255), and the outcome variable was coded as 1 = unmet need and 0 = otherwise. The category coded as 1 included both unmet rehabilitation needs during treatment and after treatment. The same explanatory variables were included in the model: the child's diagnosis (with spinal cord tumor and CNS tumor categories were merged), age at diagnosis and primary treatment center.

The results of this analysis (Table 4) suggest that a child's age at diagnosis did not influence the non-provision of care; however, both diagnosis and treatment center were significant factors. The highest risk (odds ratio) of not receiving rehabilitation care was reported among parents of children with lymphoma (the reference category). For children with ALL, the risk of not receiving care was 76% lower, for children with CNS tumors 81% lower, and for children with bone tumors even 84% lower. For other diagnoses, the risk of not receiving rehabilitation care did not differ from the reference category. Different diagnoses are therefore associated with varying levels of risk of unmet rehabilitation needs despite its necessity.

**Table 4:** Binary logistic regression model for predicting unmet rehabilitation needs.

Predictor	Category	β	S.E.	*p*-value	OR	95% CI for OR
Diagnosis	Lymphoma (ref.)			0.022		
	ALL	−1.435	0.629	0.023	0.238	(0.07–0.82)
	CNS tumor	−1.676	0.622	0.007	0.187	(0.06–0.63)
	Bone tumors	−1.810	0.788	0.022	0.164	(0.04–0.77)
	Other	−0.732	0.595	0.219	0.481	(0.15–1.54)
Age at diagnosis	0–6 years (ref.)			0.404		
	7–12 years	−0.067	0.382	0.860	0.935	(0.44–1.97)
	13–19 years	0.537	0.505	0.288	1.710	(0.64–4.62)
Treatment center	Brno (ref.)			0.012		
	Motol	−1.049	0.375	0.005	0.350	(0.17–0.73)
	Other	−0.045	0.559	0.936	0.956	(0.32–2.86)
Constant		0.320	0.662	0.564	1.465	(0.40–5.37)

*N* = 255 parents of children who needed rehabilitation care; ref., reference category; β, regression coefficient; S.E., standard error; OR, odds ratio; CI, confidence interval; statistical significance set at *p* < 0.05. Omnibus test of model coefficients was statistically significant; Nagelkerke R^2^ = 12.3%.

The results further show that the risk of not receiving care is statistically lower in Motol compared to Brno by 65%. These results can be interpreted in the context of the previous model, which showed that children treated at Motol also have higher awareness of their entitlement to rehabilitation care. In any case, it is evident that Motol places greater emphasis both on awareness of entitlements to rehabilitation care as well as on the provision of the care itself.

These quantitative findings were supported by qualitative data. When parents of children during active treatment were asked to describe barriers to receiving rehabilitation care despite expressing a desire for it, the theme consistently mentioned was failure of the healthcare system to arrange appropriate services, manifesting primarily as a lack of information regarding available services. Selected quotations:

“I didn't know about it, that it was a possibility. Then I searched for it myself through the scars, I know how important it is to take care of them.”

“No one offered us this option, nor did anyone provide any recommendation.”

or insufficient intensity of care provided. Selected quotations:

“Rehabilitation was provided, but to an absolutely minimal extent. Over the course of five months, they came to see him a total of about five times. Nothing more.”

“I am not sure where the problem was, but during the period of intensive treatment a rehabilitation nurse came to see us approximately four times. . We were not aware that we were actually entitled to this care, and this was not communicated to us.”

“I attended about three sessions with my son, during which he performed various exercises, but afterwards no one continued to follow up, even though my son needed rehabilitation.”

Among parents of children after treatment, the predominant barriers were similarly informational in nature, with an additional burden placed on families who reported having to independently arrange rehabilitation services themselves. Selected quotations:

“We had to find out everything on our own… After being discharged from intensive treatment, we were left feeling helpless and essentially came across everything by chance. At the time, we were very distressed that the follow-up care after treatment was not sufficient.”

“Reluctance, lack of expertise, and fear of treating a pediatric oncology patient.”

#### 3.2.3 Chronicity of rehabilitation need and its impact on families

**Figure 4:**
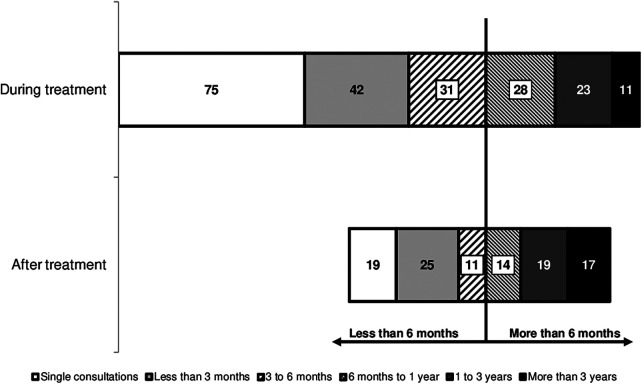
Duration of rehabilitation services required by pediatric oncology patients. Data represent the reported duration of rehabilitation among patients who received physical therapy during active cancer treatment (*n* = 210) and after treatment completion (*n* = 105). Numbers within bars indicate absolute patient counts within each duration category. The vertical line separates short-term (less than 6 months) from long-term (more than 6 months) rehabilitation.

**Figure 5:**
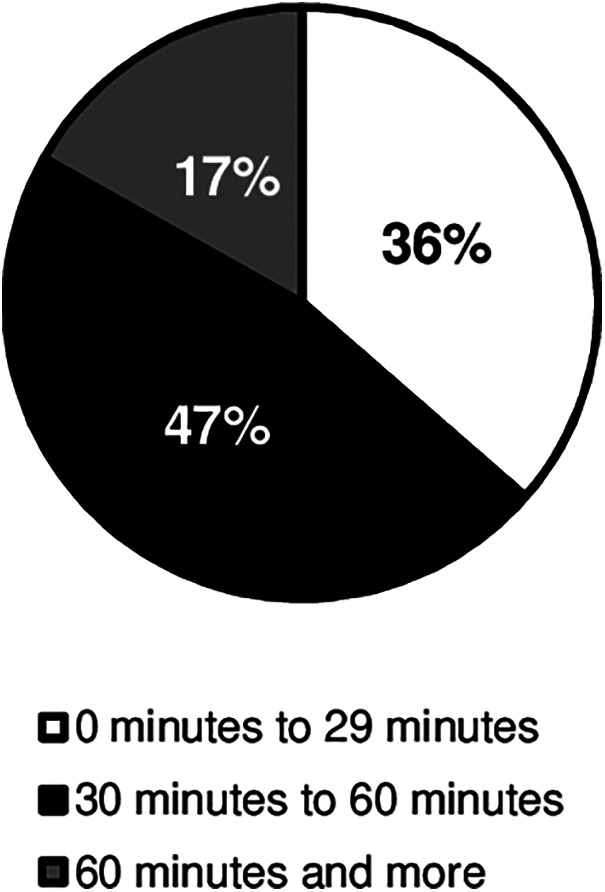
Parental willingness to commute to access specialized pediatric oncology rehabilitation. Pie chart showing the maximum travel time in minutes that parents (*n* = 231) were willing to commute to secure specialized physical therapy for their child.

Among children who received rehabilitation during active treatment, 29.5% required therapy for more than six months; this proportion rose markedly to 47.6% among those receiving post-treatment rehabilitation (Figure 4). This shift underscores that rehabilitation in pediatric oncology represents a sustained, long-term commitment rather than a time-limited intervention.The sustained nature of this rehabilitation need was mirrored in parental commitment to securing appropriate care: despite documented gaps in local service availability, 83.2% of parents expressed willingness to commute up to 60 min to access specialized pediatric oncology rehabilitation (Figure 5).

Qualitative data provided further insight into the structural barriers preventing families from accessing rehabilitation services closer to home. Among parents of children during active treatment, the predominant reasons for not utilizing local rehabilitation services were the current use of hospital-based rehabilitation or the complete absence of available services within their geographic region. Selected quotation:

“Despite all our efforts, we were unable to find any physiotherapist in our city and the surrounding area who was willing to accept a child with an oncological diagnosis.”

Among parents of children after treatment completion, barriers were considerably more heterogeneous, encompassing time constraints, regional unavailability of care, repeated hospitalizations, and reported reluctance among rehabilitation professionals to accept patients with a cancer diagnosis. Selected quotations:

“Fear, unwillingness, ignorance, limited scheduling flexibility”.

“If the care had been offered together with an explanation of why it was needed, we would have used it. The point is not only to address acute problems, but also to prevent complications that may gradually arise as a consequence of the treatment.”

“We cannot find a physiotherapist who would accept an oncology patient.”

Taken together, the qualitative findings reveal that the access gap identified in the quantitative data is not attributable to a lack of parental motivation. Rather, families consistently reported two principal barriers: insufficient information about available rehabilitation options, and difficulties identifying local providers willing to accept children with a cancer diagnosis. Among parents of children after treatment completion, these barriers were further compounded by the need to independently arrange services with limited guidance from healthcare providers.

Selected quotations:

“I had to request everything myself and after treatment ended I searched for rehabilitation professionals on my own. No option for care during the maintenance phase was ever offered to me.”

“This option was not offered to me at all. I had to arrange everything on my own.”

Collectively, these findings point to a pattern of barriers that are systemic rather than individual in nature — encompassing reported gaps in information provision, difficulties accessing local rehabilitation providers, and the burden placed on families to independently navigate care. The following section examines these barriers from the perspective of rehabilitation professionals themselves.

### 3.3 Healthcare professional perspectives

#### 3.3.1 Education gap and clinical exposure

**Figure 6:**
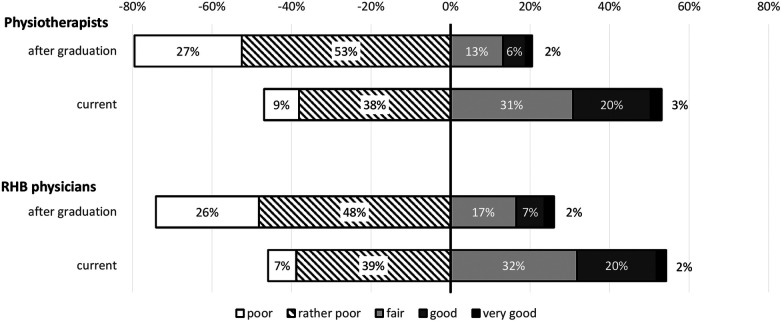
Self-assessed knowledge level in pediatric oncology rehabilitation among physiotherapists and rehabilitation physicians after graduation (retrospectively assessed) and in current practice. Diverging stacked bar chart showing the distribution of responses among physiotherapists (*n* = 318) and rehabilitation physicians (*n* = 85) regarding their self-assessed knowledge level in pediatric oncology rehabilitation. Negative values represent responses rated worse than fair, whereas positive values represent responses rated fair or better. Self-assessed knowledge levels were significantly higher in current practice compared to after graduation among both physiotherapists (*χ*²(16) = 137.452, *p* < 0.001, Cramér's *V* = 0.329) and rehabilitation physicians (*χ*²(16) = 55.890, *p* < 0.001, Cramér's *V* = 0.405). RHB physicians, rehabilitation physicians.

A critical education gap was identified among participating healthcare providers. A total of 80% of physiotherapists and 74% of rehabilitation physicians rated their knowledge of pediatric oncology rehabilitation upon graduation as insufficient, selecting “poor” or “rather poor” on a five-point scale. Although professionals rate their knowledge in current practice as statistically significantly better compared to that reported upon graduation, low levels of self-assessed knowledge persist among almost half of them, with 47.0% of physiotherapists and 46.0% of rehabilitation physicians continuing to report insufficient expertise in this field (Figure 6).

When asked specifically about their ability to recognize pediatric oncology “red flags” during rehabilitation — such as newly onset fatigue, unexpected pallor, or asymmetrical weakness — a substantial knowledge gap was identified. Over half of the physiotherapists (51.8%) and 41.0% of rehabilitation physicians rated their knowledge of these acute warning signs as either “rather insufficient” or “completely insufficient”, while only 18.6% of physiotherapists and 29.0% of physicians felt confident in this area.

This educational deficit is compounded by extremely limited clinical exposure. The vast majority of community-based professionals rarely encounter pediatric oncology patients: 71.3% of physiotherapists and 52.0% of rehabilitation physicians reported having treated four or fewer such patients over their entire career, with 37.3% of physiotherapists and 30.0% of physicians reporting zero cases. When professionals do encounter these patients, they most frequently present with neuro-oncological conditions—reported by 64.2% of physiotherapists and 68.6% of rehabilitation physicians -, followed by hematological malignancies and bone tumors. The timing of interventions also differed between professional groups: rehabilitation physicians were most often involved in care both during and after oncological treatment (57.1%), while physiotherapists primarily encountered these patients in the post-treatment survivorship phase (44.2%). Together, low case volume, high clinical complexity, and limited targeted education characterize the professional landscape of community-based pediatric oncology rehabilitation in the Czech Republic.

#### 3.3.2 Willingness to accept pediatric oncology patients: predictors and barriers

To further examine the determinants of professional engagement, binary logistic regression was conducted to identify predictors of physiotherapists' willingness to accept pediatric oncology patients during active treatment (Table 5). This analysis was limited to physiotherapists only, as the rehabilitation physician sample (*n* = 85) was insufficient for reliable regression modelling. Predictor variables included gender, years of pediatric physiotherapy practice, prior training in pediatric oncology, self-assessed knowledge of pediatric oncology, type of workplace and region of workplace. We also controlled for number of pediatric oncology patients previously treated, but this variable was not included in the model, because it was not statistically significant, but correlated with years of pediatric practice and type of workplace. Results are reported as odds ratios (OR) with 95% confidence intervals. Statistical significance was set at *p* < 0.05.

**Table 5:** Binary logistic regression predicting physiotherapists’ willingness to accept pediatric oncology patients during active treatment.

Predictor	Category	β	S.E.	*p*-value	OR	95% CI for OR
Gender	Female (ref.)					
	Male	0.183	0.490	0.709	1.200	(0.46–3.13)
Years of pediatric practice	Continuous	0.098	0.071	0.163	1.103	(0.96–1.27)
Training in pediatric oncology	No (ref.)					
	Yes	0.644	0.365	0.078	1.904	(0.93–3.89)
Self-assessed knowledge	Insufficient (ref.)					
	Sufficient	1.032	0.280	0.000	2.807	(1.63–4.84)
Workplace type	Other (ref.)					
	University hospital	1.294	0.536	0.016	3.648	(1.27–10.47)
	General hospital	0.213	0.434	0.624	1.237	(0.53–2.90)
Region	Moravia (ref.)					
	Prague	0.103	0.396	0.795	1.108	(0.51–2.41)
	Bohemia	0.695	0.318	0.029	2.003	(1.08–3.72)
	South Moravia (Brno)	0.424	0.506	0.402	1.529	(0.57–4.12)
Constant		−0.731	0.420	0.082	0.481	(0.21–1.10)

*N* = 318 physiotherapists; ref., reference category; β, regression coefficient; S.E., standard error; OR, odds ratio; CI, confidence interval; statistical significance set at *p* < 0.05. Omnibus test of model coefficients was statistically significant; Nagelkerke R^2^ = 19%.

While gender, years of pediatric practice, and training in pediatric oncology were not statistically significant, self-assessed knowledge emerged as strong predictor: physiotherapists rating their pediatric oncology knowledge as sufficient were almost 3 times more likely to express willingness compared to those rating it as insufficient (OR = 2.81, *p* < 0.001). Workplace setting and regional location were also statistically significant predictors.

The results indicate higher willingness to accept pediatric oncology patients during active treatment among physiotherapists working in university hospitals compared to those in other settings (OR = 3.65, *p* = 0.016), as well as higher willingness among physiotherapists in Bohemia compared to those in Moravia (OR = 2.003, *p* = 0.029). This may reflect weaker infrastructure or lower levels of experience outside large hospitals and central regions.

Regarding the qualitative analysis of barriers perceived by physiotherapists, two principal themes emerged. The first, characterized as a lack of professional expertise, encompassed insufficient clinical experience, inadequate specialized education, and apprehension regarding this patient population. Selected quotation:

“Many of my colleagues are concerned about whether they will be able to work with these patients.”

The second theme, characterized as healthcare system limitations, primarily included insufficient time allocated per patient, prolonged waiting times, and concerns about patient safety in the context of treatment-related immunosuppression. Selected quotation:

“My outpatient clinic is located within a polyclinic — there is a risk of infection, most commonly respiratory.”

Among rehabilitation physicians, reported barriers were considerably more heterogeneous, ranging from insufficient workplace capacity and lack of necessary technical equipment to limited clinical experience and absence of interdisciplinary collaboration. Selected quotation:

“The absence of other specialists within multidisciplinary cooperation”.

These findings suggest that the reluctance of community-based professionals to accept pediatric oncology patients does not stem from a lack of motivation, but rather from a substantial deficit in targeted education and clinical exposure, which a structured competence network may be well positioned to address.

### 3.4 Stakeholder perspectives on RehaSÍŤ: interest and perceived benefits

**Figure 7:**
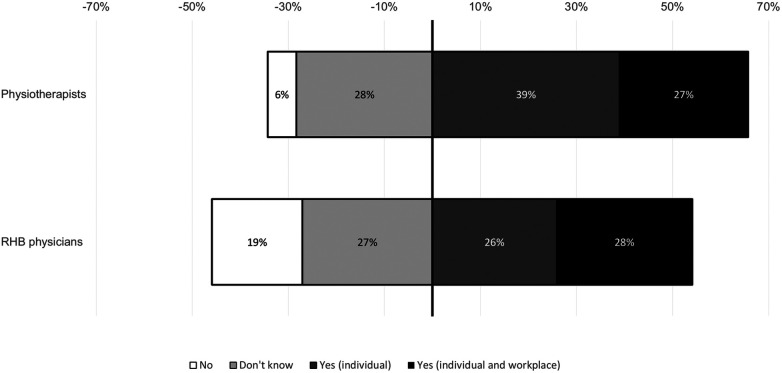
Interest in joining the RehaSÍŤ rehabilitation network among physiotherapists and rehabilitation physicians. Diverging stacked bar chart showing the distribution of responses among physiotherapists (*n* = 318) and rehabilitation physicians (*n* = 85) regarding interest in joining the RehaSÍŤ rehabilitation network. The 0% line marks the boundary between “Yes” responses (positive values) and the remaining response categories. RHB physicians, rehabilitation physicians.

**Figure 8:**
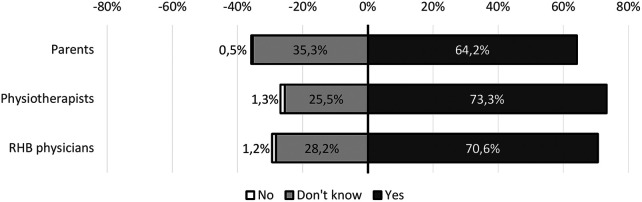
Perceived added value of the RehaSÍŤ network among parents, physiotherapists, and rehabilitation physicians. Diverging stacked bar chart showing the distribution of responses among parents (*n* = 394), physiotherapists (*n* = 318), and rehabilitation physicians (*n* = 85) regarding whether an optimally functioning RehaSÍŤ network would bring positive benefits or added value. The 0% line marks the boundary between “Yes” responses (positive values) and the remaining response categories. RHB physicians, rehabilitation physicians.

Despite these barriers, 65.7% of physiotherapists and 54.0% of rehabilitation physicians expressed interest in joining RehaSÍŤ (Figure 7), and a substantial majority across all three stakeholder groups anticipated positive benefits from an optimally functioning network (Figure 8).

This high level of cross-stakeholder endorsement provided the quantitative foundation for the qualitative exploration of perceived benefits presented below.

Potential benefits of the RehaSÍŤ project for key stakeholders were further explored using responses to open-ended questions and qualitative analysis. All three primary stakeholder groups were asked to describe the perceived benefits of the project, and three main themes emerged consistently across groups. The first theme, networking, encompassed distinct yet complementary perspectives across stakeholders. Parents predominantly described benefits related to access to a comprehensive contact database and improved connectivity among healthcare professionals. Selected quotation:

“We would be able to easily find contacts for physiotherapists in the surrounding area”

Healthcare professionals similarly highlighted the value of a shared contact database, while also emphasizing opportunities for interdisciplinary collaboration and experience sharing: “*The possibility of referring parents of patients to relevant contacts”*.

The second theme, access to information, included references to readily available, up-to-date information, clinical guidelines, and the consolidation of relevant resources into a single accessible platform. Selected quotation:

“Easier navigation through the tangle of information and rehabilitation facilities”.

The third theme, enhancement of rehabilitation services, also reflected differing priorities across stakeholder groups. For parents, this theme primarily encompassed improved availability of services, greater perceived professional expertise of healthcare providers, and more supportive attitudes toward patients and families. Selected quotation:

“In our close social circle we have a child with an oncology diagnosis who is in need of rehabilitation services. We know that the family would appreciate a shorter commute to access these services.”

For healthcare professionals, it predominantly concerned continuing professional education and improved continuity of care across treatment phases. Selected quotation:

“Enhanced integration of rehabilitation care between oncology centers and peripheral regions, with continuity of care in the patient's home region.”.

Together, the high level of stakeholder interest and the convergent themes of perceived benefit suggest that the conditions for successful network implementation are present across all three key groups.

## 4 Discussion

### 4.1 Informational deficit as a driver of the access gap

A critical and largely overlooked finding of this study is that informational barriers precede and compound the access gap: only 51.0% of parents reported being explicitly informed about their child's eligibility for rehabilitation services, despite 64.2% expecting such information as a matter of course. This pattern is not unique to the Czech context. Internationally, parents of children with cancer consistently report adequate information about diagnosis and treatment, while simultaneously describing substantial gaps in information about supportive care services and rehabilitation pathways — particularly at key transitions. In a multinational study, 92% of parents wanted more information about what to expect when their child completed treatment, yet most reported that relevant discussions did not take place (23). Swedish qualitative evidence similarly documents that parents feel “abandoned” at key milestones, are forced to actively seek information themselves, and describe the experience of navigating supportive care as “learning a new language” (24). These patterns suggest that informational deficits around rehabilitation are a structural rather than incidental feature of pediatric oncology care — one that our data confirm is present also in the Central European context.

The informational asymmetry we identified — whereby parents of children with CNS tumors were eight times more likely to report having been informed than parents of children with lymphomas (OR = 8.02, *p* < 0.001), and parents treated at Motol University Hospital twice as likely to report awareness compared to those treated in Brno (OR = 2.25, *p* = 0.006) — points consistently to structural rather than individual determinants of information provision. Children with CNS tumors present with neuromotor deficits that are immediately apparent, making rehabilitation referral intuitive; children with lymphomas, despite significant treatment-related late effects, may not trigger the same reflexive referral pathway. Crucially, both the diagnostic and the institutional effect suggest that information provision depends on the visibility of functional impairment and on center-specific practices rather than on universal clinical protocols. This interpretation is supported by international evidence: a US survey found that 76% of hospitals did not have an established pediatric oncology rehabilitation program (25), qualitative evidence from the UK documents a lack of standard practices for transition support with practices rarely recorded in writing (26), and Canadian healthcare professionals similarly identified the absence of standardized referral processes as a key structural barrier (27). Together, these findings suggest that diagnostic and institutional disparities in rehabilitation awareness are two manifestations of the same underlying problem: the absence of systematically embedded information pathways in pediatric oncology care. Our qualitative data reinforce this interpretation — families who were not proactively directed to services frequently had to independently seek and arrange rehabilitation care, describing the experience as navigating a fragmented system without guidance. Addressing this deficit requires moving beyond individual provider communication toward embedded, institutionalized pathways — including proactive referral systems, case management roles, and standardized transition programs — that make rehabilitation information a routine and equitable component of oncological care (26).

### 4.2 The access gap: scope, diagnosis, and chronicity

Our findings confirm a substantial and widening gap between the need for rehabilitation and its actual provision across both phases of pediatric oncology care. An overall unmet rehabilitation need rate of 10.4% during active treatment — meaning approximately one in ten children who required rehabilitation did not receive it — increased to 13.3% following treatment completion. While direct prevalence estimates across the entire pediatric oncology spectrum remain scarce (28), the pattern we identified is consistent with international evidence of systematic underutilization. A large cohort study involving over 9,000 childhood cancer survivors found that only 9.2% utilized physical therapy — a rate no higher than their unaffected siblings — despite 20% experiencing physical function impairments, underscoring that clinical need does not automatically translate into service utilization (29). Although more recent claims-based data suggest an encouraging upward trend in physical therapy utilization for specific diagnoses such as acute lymphoblastic leukemia (30), our data indicate that substantial gaps persist, particularly in the post-treatment survivorship phase.

When stratified by diagnosis, spinal cord tumor patients showed the highest descriptive unmet need rates across both phases (25.0% during treatment, 28.6% post-treatment), followed by lymphoma survivors (14.3% and 22.6%, respectively), while CNS tumor patients reported no unmet needs post-treatment (0%). Given the very small absolute numbers in the spinal cord tumor subgroup (*n* = 3 and *n* = 2, respectively), these percentages should be interpreted with caution. Binary logistic regression confirmed lymphoma as the diagnosis associated with the highest statistically robust risk of not receiving care, with ALL, CNS tumor, and bone tumor patients showing 76%, 81%, and 84% lower risk, respectively (Table 4). Overall, these unmet need rates remain substantially lower than those reported in other national contexts — unmet rehabilitation needs have been documented in up to 86% of pediatric brain tumor survivors in Norway (31) — consistent with the broader observation that unmet need reflects the structural capacity of healthcare systems as much as diagnosis-specific factors (28).

Perhaps the most clinically significant finding of this study is the marked increase in the chronicity of rehabilitation need following treatment completion. Among children who received rehabilitation during active treatment, 29.5% required therapy for more than six months — a proportion that rose to 47.6% among those receiving post-treatment rehabilitation. This shift reflects a well-documented trajectory in which certain functional deficits not only persist but may worsen during the survivorship phase. In survivors of acute lymphoblastic leukemia, functional exercise capacity remains significantly impaired at a median of more than five years after completing chemotherapy, even as general motor performance improves (32). A systematic review and meta-analysis further found that deficits in skeletal muscle mass become progressively greater with increasing time since treatment completion, suggesting a long-term divergence between survivors and their healthy peers that worsens rather than resolves (33). Chronic fatigue — defined as severe fatigue lasting six months or longer — affects 23.6% of long-term survivors (34), representing a further dimension of rehabilitation need that extends well beyond the acute treatment phase. These findings collectively underscore that the 47.6% of children requiring long-term rehabilitation post-treatment identified in our cohort represent not an anomaly, but an expected and chronically underserved population.

### 4.3 The burden on families

The willingness of 83.2% of parents in our cohort to commute up to 60 min to access specialized pediatric oncology rehabilitation is a finding that warrants careful interpretation. Rather than reflecting an acceptable model of care delivery, it signals the absence of accessible alternatives — a distinction with significant implications for family well-being and health equity. This interpretation is supported by our qualitative data, in which parents described repeated failed attempts to locate willing rehabilitation providers in their home regions, and by the finding that barriers to local care included not only geographic unavailability but also the reluctance of community professionals to accept children with an oncological diagnosis.

The contrast with findings from the Netherlands is instructive. In the KinderOncoNet needs assessment, 80% of Dutch parents were willing to travel 15 to 30 min to access a local pediatric physiotherapist with oncology expertise (14) — a substantially shorter distance than the 60 min threshold accepted by the majority of parents in our cohort. This divergence likely reflects differences in the availability of community-based oncology rehabilitation between the two countries rather than differences in parental motivation, and underscores the degree to which Czech families are currently forced to compensate for structural gaps through personal effort and sacrifice.

Relying on families to overcome geographic barriers through repeated long-distance travel places an immense and often unsustainable burden on them. Systematic reviews consistently identify travel distance and the physical demands of long journeys as primary drivers of exhaustion and life disruption in pediatric oncology (35). Travel-related out-of-pocket expenses, combined with the necessity for parental work disruptions — which affect the vast majority of caregiving families — precipitate significant economic impacts and long-term financial instability (36–38). Consequently, the remarkable willingness of parents in our cohort to commute should not be interpreted by policymakers as evidence of a functioning system, but rather as a symptom of a critical lack of accessible, community-based rehabilitation infrastructure.

### 4.4 The workforce crisis: education, exposure, and willingness

The access gap and family burden documented in the preceding sections cannot be understood in isolation from the profound workforce crisis identified among community-based rehabilitation professionals. Our data reveal that 80% of physiotherapists and 74% of rehabilitation physicians rated their knowledge of pediatric oncology rehabilitation as insufficient upon graduation — a deficit that persists for nearly half of them even in current practice. This educational vacuum is not unique to the Czech Republic. A US survey of rehabilitation practitioners found that 65% received no education in pediatric cancer rehabilitation during their training, resulting in the absence of dedicated programs in 76% of surveyed hospitals (25). The pattern suggests a global systemic failure to integrate pediatric oncology rehabilitation into professional education curricula.

This lack of specialized training is compounded by extremely limited clinical exposure. In our cohort, 71.3% of physiotherapists and 52.0% of rehabilitation physicians reported having treated four or fewer pediatric oncology patients over their entire career, with 37.3% of physiotherapists having never treated one. The consequent gap in practical experience is clinically significant: when community providers do encounter these patients, they most frequently present with neuro-oncological sequelae — reported by 64.2% of experienced physiotherapists and 68.6% of physicians — representing precisely the most complex clinical presentations. This mirrors findings from the Netherlands, where local pediatric physiotherapists similarly reported seeing none to four such children across their careers, with 40% citing insufficient knowledge as a primary barrier to high-quality care (14). The combination of high clinical complexity, low case exposure, and insufficient evidence-based guidelines for safe exercise parameters during active treatment creates a pervasive environment of clinical uncertainty (39, 40).

The consequences of this uncertainty are reflected in our qualitative data, where physiotherapists identified two principal barriers: insufficient professional expertise and healthcare system limitations — including concerns about patient safety in the context of immunosuppression. Critically, over half of physiotherapists (51.8%) and 41.0% of rehabilitation physicians rated their knowledge of acute oncological red flags as insufficient — a safety concern that our regression analysis helps contextualize. Physiotherapists who rated their knowledge as sufficient were nearly three times more likely to express willingness to accept pediatric oncology patients during active treatment (OR = 2.81, 95% CI 1.63–4.84, *p* < 0.001), confirming that the primary barrier is not motivation but perceived competence. This finding aligns with broader evidence from the Central and Eastern Europe region: a survey of physical therapists across Eastern Europe — including the Czech Republic — found that therapists in this region less frequently adjusted their therapy to the severity of patient disability compared to colleagues in other European regions (40% vs. 65%, *p* = 0.009), a proxy measure suggesting lower confidence in managing complex clinical presentations in this region, albeit in a different diagnostic context (41).

Workplace setting emerged as the second strongest independent predictor of willingness: physiotherapists working in university hospitals were 3.6 times more likely to express willingness than those in other settings (OR = 3.65, 95% CI 1.27–10.47, *p* = 0.016). This institutional effect likely reflects the differential access to specialist mentorship, oncology case volume, and interdisciplinary collaboration that university hospital environments provide. In Poland, physiotherapist density in large urban counties exceeds that in small rural counties by nearly 40% (42) — a structural gradient that likely perpetuates differential exposure to complex cases and specialist training opportunities across practice settings. A similar pattern of infrastructure-dependent confidence likely underlies the regional variation observed within our own data. Notably, neither gender, years of clinical practice, nor prior exposure to pediatric oncology patients predicted willingness — underscoring that accumulated general experience cannot substitute for targeted oncology-specific training, and that the locus of intervention must be education and institutional environment rather than individual professional development alone.

### 4.5 Toward a structured competence network: the RehaSÍŤ model

The convergence of findings across this study — a persistent access gap, chronically underserved families, and a workforce lacking confidence rather than motivation — points toward a single systemic solution: a structured competence network bridging specialized tertiary expertise with community-based care. The feasibility of such a network is supported by our stakeholder data, though results require careful contextual interpretation. While the vast majority of respondents anticipated positive benefits from an optimally functioning RehaSÍŤ network (73.3% of physiotherapists, 70.6% of rehabilitation physicians, 64.2% of parents), the substantial “don't know” responses (25%–35% across groups) reflect unfamiliarity with the concept rather than ambivalence — a pattern mirroring a Czech needs assessment for ParkinsonNet implementation, which concluded that conditions for network development were present but infrastructure had yet to be built (43). This stands in instructive contrast to the Dutch KinderOncoNet survey, where 93% of physiotherapists and 97% of parents endorsed an equivalent network (14) — a difference likely reflecting the Netherlands' established tradition of disease-specific rehabilitation networks rather than greater intrinsic enthusiasm.

International evidence supports the transition to shared-care models in pediatric oncology. Hub-and-satellite structures have been shown to reduce travel burden and improve patient satisfaction without compromising clinical safety (44, 45), and evidence from shared-care survivorship models demonstrates that structured specialist collaboration more than doubles community provider confidence — primary care providers’ comfort in managing childhood cancer survivors rose from 35.6% when acting independently to 75.6% when working alongside a pediatric oncologist (46). In a shared-care follow-up pilot, 98% of community physicians were willing to collaborate and 82% reported satisfaction with specialist-provided information (47), suggesting that the latent workforce capacity identified in our data is readily mobilizable when appropriate structural supports are in place.

It is important to acknowledge that robust evidence directly linking rehabilitation-focused competence networks to measurable improvements in community provider readiness and patient access remains limited (27). The RehaSÍŤ model will therefore need to incorporate prospective evaluation from its inception — measuring provider confidence, network participation, rehabilitation access rates, and functional outcomes — to generate the evidence base that the field currently lacks. While integrating rehabilitation into standard oncology pathways remains a global challenge (48), the strong stakeholder endorsement documented here, combined with the availability of international models to adapt, suggests that the Czech Republic is well positioned to develop a sustainable, equitable model of community-based pediatric oncology rehabilitation that could serve as a blueprint for similar healthcare systems in the region.

### 4.6 Limitations

Several limitations of this study warrant consideration. First, the study employed convenience sampling with voluntary self-selection through online recruitment channels, which may have introduced participation bias. Parents more actively engaged in their child's care and healthcare professionals with a pre-existing interest in pediatric oncology rehabilitation were more likely to respond, potentially overestimating both parental demand and professional awareness of rehabilitation needs. Furthermore, participants recruited via online platforms may differ systematically from non-respondents in terms of digital literacy, personal engagement with the topic, or access to relevant information networks. These factors may have introduced self-selection bias and should be considered when interpreting the findings and their generalizability to the broader population of Czech families and rehabilitation professionals. Second, the cross-sectional design precludes causal inference — while our data demonstrate associations between variables such as self-assessed knowledge and willingness to treat, the directionality of these relationships cannot be established. Third, data from parents whose children completed treatment several years prior rely on retrospective recall, which may be subject to memory bias regarding the timing, intensity, and adequacy of rehabilitation services received. Fourth, the small absolute numbers of patients within several diagnostic subgroups — most notably spinal cord tumors (*n* = 12) — limit the reliability of subgroup-level comparisons and should be interpreted with caution. Fifth, several regression analyses — particularly those involving comparisons between diagnostic subgroups — yielded wide confidence intervals and large odds ratios, indicating reduced statistical precision and greater uncertainty regarding the magnitude of the observed associations. This is an inherent consequence of the relatively small sample sizes within individual diagnostic categories, reflecting the rarity of specific childhood cancer diagnoses in the general population. The study aimed to include all eligible participants within the data collection period; however, the number of children in some diagnostic groups remained limited despite these efforts. Subgroup-level regression estimates should therefore be considered exploratory and interpreted with caution. Future studies with larger samples, ideally involving multiple centres or national registry linkage, are needed to confirm and refine these findings. Sixth, the regression analysis examining predictors of willingness to treat was limited to physiotherapists only, as the rehabilitation physician sample (*n* = 85) was insufficient for reliable modelling, precluding direct comparison of predictors across professional groups. Seventh, all data are self-reported, and self-assessed knowledge in particular may be subject to systematic bias — professionals with limited exposure may either underestimate or overestimate their competence. Eighth, this study was conducted exclusively in the Czech Republic, within a specific healthcare system characterized by the coordinating role of rehabilitation physicians. The generalizability of findings to countries with different healthcare structures and financing models should therefore be considered with caution. Finally, the perspectives of children and adolescents with cancer themselves were not captured in this study. Parents served as proxies, and it remains unknown to what extent their responses reflect discussions held with their children about rehabilitation experiences and needs. Future studies should seek to directly incorporate the voices of pediatric patients and survivors.

### 4.7 Conclusions

This study provides a comprehensive, multi-perspective assessment of the rehabilitation landscape in pediatric oncology in the Czech Republic. The findings confirm a substantial and widening gap between rehabilitation need and its actual provision: approximately one in ten children with cancer did not receive needed rehabilitation during active treatment, a proportion that increased following treatment completion. Informational deficits — with only half of parents explicitly informed of their child's rehabilitation entitlement — emerged as a primary structural driver of this access gap, compounded by significant institutional and diagnostic disparities in information provision. The chronicity of rehabilitation need, with nearly half of children requiring therapy for more than six months after treatment completion, underscores that pediatric oncology rehabilitation represents a sustained long-term commitment rather than a time-limited intervention.

The workforce analysis revealed that professional reluctance to treat pediatric oncology patients reflects a critical deficit in targeted education and clinical exposure rather than a lack of motivation. Self-assessed knowledge was the strongest predictor of willingness to engage — a finding with direct implications for workforce development. The high level of cross-stakeholder interest in the RehaSÍŤ network, combined with the convergent perceived benefits identified across parents, physiotherapists, and rehabilitation physicians, indicates that the structural and motivational conditions for network implementation are present. Establishing targeted educational programs and a structured competence network represents a promising and feasible pathway toward ensuring equitable, high-quality rehabilitation access for children with cancer in Central Europe and beyond.

## Data Availability

The raw data supporting the conclusions of this article will be made available by the authors, without undue reservation.
