# Supplementary material for: Bridging the gap in pediatric cancer rehabilitation care: a multi-perspective survey study
Source: Front Pediatr. 2026 Jul 7;14:1870844. doi: 10.3389/fped.2026.1870844 (PMC13385515; doi:10.3389/fped.2026.1870844)
Supplement: Supplementary file 1 [file Table1.docx]

**List of open-ended questions used for qualitative analysis**

| **Question Code** | **Target Group** | **Full Question Text (English Translation)** |
| --- | --- | --- |
| RB5 | Parents | If you were interested in receiving rehabilitation care during the intensive or maintenance phase of oncological treatment, but it was not provided to you, what was the problem? Please describe: |
| RB6 | Parents | If you were interested in receiving rehabilitation care in your region (municipality, district, county) during the intensive or maintenance phase of the oncological treatment, but it was not provided to you, what was the problem? Please describe: |
| RC5 | Parents | If you were interested in receiving rehabilitation care in your region (municipality, district, county) after completing treatment, but it was not provided to you, what was the problem? Please describe: |
| RC6 | Parents | If you were interested in receiving rehabilitation care after completing oncological treatment, but it was not provided to you, what was the problem? Please describe: |
| RD6 | Parents | Please describe what benefits or added value an optimally functioning RehaSÍŤ network would provide for you: |
| FD5 | Physiotherapists | Please describe what benefits or added value an optimally functioning RehaSÍŤ network would provide for you: |
| FB10 | Physiotherapists | What are the main barriers or limitations to providing pediatric oncological rehabilitation care at your workplace? |
| LD5 | Rehabilitation physicians | Please describe what benefits or added value an optimally functioning RehaSÍŤ network would provide for you: |
| LB8 | Rehabilitation physicians | What are the main barriers or limitations to providing pediatric oncological rehabilitation care at your workplace? |
